# Signal enhancement for two-dimensional cryo-EM data processing

**DOI:** 10.1017/S2633903X23000065

**Published:** 2023-03-09

**Authors:** Guy Sharon, Yoel Shkolnisky, Tamir Bendory

**Affiliations:** 1 School of Electrical Engineering, Tel Aviv University, Tel Aviv, Israel; 2 School of Mathematical Sciences, Tel Aviv University, Tel Aviv, Israel

**Keywords:** Cryo-electron microscopy, signal enhancement, 2D classification

## Abstract

Different tasks in the computational pipeline of single-particle cryo-electron microscopy (cryo-EM) require enhancing the quality of the highly noisy raw images. To this end, we develop an efficient algorithm for signal enhancement of cryo-EM images. The enhanced images can be used for a variety of downstream tasks, such as two-dimensional classification, removing uninformative images, constructing ab initio models, generating templates for particle picking, providing a quick assessment of the data set, dimensionality reduction, and symmetry detection. The algorithm includes built-in quality measures to assess its performance and alleviate the risk of model bias. We demonstrate the effectiveness of the proposed algorithm on several experimental data sets. In particular, we show that the quality of the resulting images is high enough to produce ab initio models of 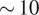 Å resolution. The algorithm is accompanied by a publicly available, documented, and easy-to-use code.

## Impact Statement

In the past few years, single-particle cryo-electron microscopy (cryo-EM) has become the state-of-the-art method for resolving the atomic structure and dynamics of biological molecules. We design an efficient algorithm to enhance the quality of the highly noisy cryo-EM experimental images. The enhanced images can be used in a wide variety of tasks in the algorithmic pipeline of cryo-EM, including two-dimensional classification, removal of uninformative images, ab initio modeling, dimensionality reduction, symmetry detection, quick assessment of the data set, and as templates for particle picking. We provide a documented Python code. The algorithm includes built-in quality measures to mitigate the risk of model bias.

## 1. Introduction

In the past few years, single-particle cryo-electron microscopy (cryo-EM) has become the state-of-the-art method for resolving the atomic structure and dynamics of biological molecules^(^^1^ ^–^^5^ ^)^. A cryo-EM experiment results in a large set of images, each corresponding to a noisy tomographic projection of the molecule of interest, taken from an unknown viewing direction. In addition, the electron doses transmitted by the microscope must be kept low to prevent damage to the radiation-sensitive biological molecules, inducing signal-to-noise ratio (SNR) levels that might be as low as –20 dB (i.e., the power of the noise is 100 times greater than the signal)^(^^6^ ^)^. The low SNR level is one of the main challenges in processing cryo-EM data sets. In particular, different tasks in the computational pipeline of cryo-EM require enhancing the quality of the highly noisy raw images. Specifically, the high-quality enhanced images can be used as two-dimensional (2D) class averages, to remove uninformative images (e.g., pure noise images, contamination), to construct ab initio models based on the common-lines property^(^^7^ ^,^^8^ ^)^,^1^ as templates for particle picking, to provide a quick assessment of the particles, for dimensionality reduction, and for symmetry detection^(^^6^ ^,^^11^ ^)^.

In this paper, we propose a new signal enhancement algorithm that quickly produces multiple enhanced images that represent different viewing directions of the molecule of interest. The algorithm begins by performing steerable principal component analysis (sPCA) that reduces the dimensionality of the data and allows rotating (steering) the images easily^(^^12^ ^)^. Next, we randomly choose a subset of the images and find their nearest neighbors based on (approximately) rotationally invariant operations. Hereafter, we refer to each image and its neighbors as a class. Then, we apply two stages for refining the classes. We first remove low-quality classes, and then also remove individual images which are inconsistent with their classes. These stages are based on inspecting the spectra of designed matrices, called synchronization matrices. The spectra of these matrices (that is, the distribution of their eigenvalues) provide a built-in quality measure to assess the consistency of each class. This is essential to support downstream tasks, such as ab initio modeling. Finally, we run an expectation–maximization (EM) algorithm for each class independently. This step aligns and averages the remaining images in each class, producing high SNR enhanced images. The different steps of the algorithm are elaborated in Section 2.

Our algorithm is inspired by and builds on the general scheme of^(^^13^ ^)^. In particular, the authors of^(^^13^ ^)^ suggest finding the rotationally invariant nearest neighbors of each image based on the bispectrum: a third-order rotationally invariant feature^(^^14^ ^)^. Then, a high-quality image is produced by aligning all neighbors and averaging. While this method works quite well in many cases, the bispectrum inflates the dimensionality of the problem, boosts the noise level (which is already high in typical data sets), and does not offer a systematic way to assess the performance of the algorithm. Section 2 elaborates on the differences between^(^^13^ ^)^ and our proposed algorithm.

Our work also shares similarities with 2D classification algorithms, which cluster the particle images and average them to produce high SNR images, dubbed class averages. 2D classification is a standard routine in all contemporary cryo-EM computational pipelines and is mostly used to remove uninformative images that are associated with low-quality class averages and to provide a quick assessment of the particles; our algorithm can be used for those tasks as well. A popular solution to the 2D classification task, implemented in the software RELION, is based on maximizing the posterior distribution of the classes, while marginalizing over the rotations and translations, using an EM algorithm^(^^15^ ^)^; we describe this methodology in more detail in Section 2.5. A large number of class averages leads, however, to high computational complexity and to low-quality results because only a few images are assigned to each class. In addition, EM tends to suffer from the “rich get richer” phenomenon (also dubbed the attraction problem): most experimental images would correlate well with, and thus be assigned to, the class averages that enjoy higher SNR. As a result, EM tends to output only a few informative classes^(^^16^ ^)^. We circumvent this phenomenon since we apply the EM to each class separately.

Another related research thread considers denoising at the image or at the micrograph level. One example of the former is denoising based on Wiener filtering^(^^17^ ^)^. A popular micrograph denoising technique is TOPAZ^(^^18^ ^)^, which is based on deep learning methods; see also^(^^19^ ^,^^20^ ^)^. However, these techniques do not harness the similarity between particle images to suppress the noise, and thus their denoising quality is limited. For example, we use the enhanced images (the outputs of our algorithm) to directly construct molecular structures at a low to intermediate resolution. As far as we know, this has not been done using the said methods.

The paper is organized as follows. Section 2 outlines our method. In Section 3, we present results on four experimental data sets. We attain high-quality images that can be used to construct ab initio models with resolutions between 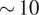 Å to 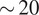 Å. Section 4 concludes the paper and delineates future work to improve the algorithm.

## 2. Method

This section describes the main steps of the proposed algorithm. A documented Python code is available at https://github.com/TamirBendory/CryoEMSignalEnhancement.

### 2.1. Preprocessing

The algorithm begins with a few standard preprocessing steps: we apply phase-flipping to approximately correct the effect of the CTF, down-sample the images to a size of 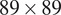 pixels, and whiten the noise in the images. The down-sampling has a minor effect on the nearest neighbors search, and is thus used to accelerate the running time of the algorithm. Then, we further reduce the dimension of the images using sPCA, which learns a steerable, data-driven basis for the data set^(^^12^ ^)^. Under this basis, the 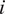 th image can be approximated by a finite expansion
(2.1)

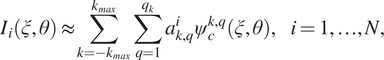

where 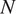 is the number of images in the data set, 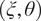 are polar coordinates, 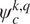 are the sPCA eigenfunctions, 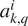 are the corresponding coefficients, 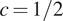 is the bandlimit of 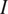 , and 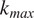 and 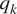 are determined as described in^(^^12^ ^)^. Remarkably, under this representation, an in-plane rotation translates into a phase shift in the expansion coefficients
(2.2)

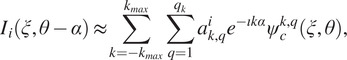

where 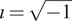 is the imaginary unit, and, for real-valued images, a reflection translates into conjugation
(2.3)

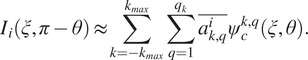

The sPCA dramatically reduces the dimensionality of the images. We use 500 sPCA coefficients to represent the images. Henceforth, with a slight abuse of notation, we refer to the vector of sPCA coefficients of the 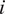 th image 
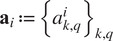
 as the image.

We mention that the final stage of the algorithm, the EM step, uses the raw images, which are not affected by these preprocessing steps.

### 2.2. Nearest neighbor search

Next, we randomly choose 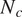 images 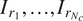 from the data set and find the 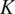 nearest neighbors of each image. The underlying assumption is that the nearest neighbors arise from similar viewing directions. We refer to an image and its 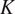 neighbors as a *class.*

The nearest neighbors search is based on a correlation measure which is approximately invariant under in-plane rotations and reflection. Let 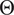 be a predefined set of 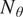 angles; we typically use 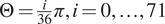 so that 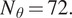 We define the approximately invariant correlation, between two images 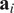 and 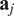 , by
(2.4)

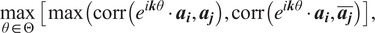

where
(2.5)

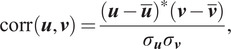

and where 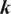 is a radial frequency vector,  denotes element-wise product, and 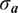 is the standard deviation of a vector 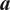 . The nearest neighbors of the 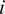 -th image are chosen as the 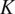 images with the highest correlation (2.4).

The nearest neighbors search requires computing 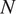 correlations for each of the 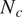 selected images, resulting in a total of 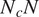 correlations. For each image, the correlations can be computed using a couple of matrix multiplications using established linear algebra libraries. The computational complexity of this stage is governed by the multiplication of matrices of size 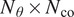 and 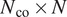 , where 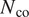 is the number of sPCA coefficients. For the experiments in Section 3, this stage took less than a minute.

Two comments are in order. First, the approximately invariant correlation can be, in principle, replaced with invariant polynomials called the bispectra, giving rise to analytical rotationally invariant features^(^^13^ ^,^^14^ ^)^ or approximately rotationally and translationally invariant features^(^^21^ ^)^ However, the dimension of the bispectrum far exceeds the dimension of the image, and thus we preferred to use the more direct expression of (2.4). Second, we choose the 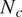 images at random in order to cover different viewing directions. In a future work, we hope to replace this random strategy with a deterministic technique that finds a set of images covering all viewing directions.

We next describe a method to rank and remove low-quality classes resulting from our random sampling strategy. This method provides a built-in measure of the quality of the classes, and thus of the enhanced images.

### 2.3. Sorting the classes

Until now, we have randomly chosen a set of images 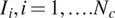 , and found 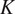 nearest neighbors per class. However, since the images 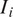 were chosen randomly, it is plausible that some of them will be uninformative in the sense that they do not have close neighbors. To discard uninformative images and their classes, we aim to rank the classes according to their quality.

We define a good class as a class where all of its members were taken from a similar viewing direction, up to an in-plane rotation and, possibly, a reflection. For each pair of images in the class, we compute the most likely relative in-plane rotation and reflection; this is a by-product of computing the correlations in (2.4) so no additional computations are required. We denote the estimated relative rotation angle between the 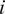 th and 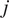 th members of the 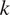 th class by 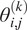 . If no reflection is involved, the relative rotation can be represented by a 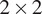 rotation matrix
(2.6)

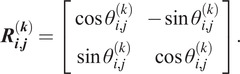

If the pair of images are also reflected, then
(2.7)

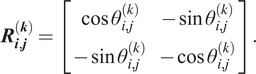

We then construct a Hermitian block matrix 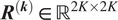



(2.8)

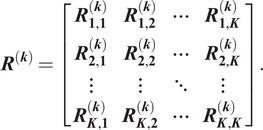

The matrix 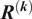 is a synchronization matrix over the dihedral group^(^^22^ ^)^. If indeed all 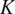 class members are the same image up to an in-plane rotation and, possibly, a reflection (namely, an element of the dihedral group), then 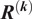 is of rank two. In other words, only the first two largest eigenvalues 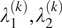 of 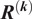 are nonzero. In practice, since the images were not taken precisely from the same viewing direction, and because of the noise, the matrix is not of rank two. Therefore, as a measure of the quality of this class, it is only natural to compute how “close” is 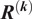 to a rank two matrix, namely,
(2.9)

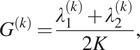

where we note that 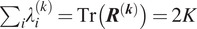 . We refer to 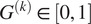 as the grade of the 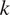 th class. We repeat this procedure for each class, and remove the classes with the lowest grades. In practice, we found that removing half of the classes yields good results. Since we need to extract the two leading eigenvalues of 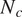 synchronization matrices, the typical computational complexity of this stage is 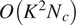 .

The empirical distributions of the eigenvalues, or the grades 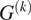 , provide a measure to assess the performance of the algorithm. This is important since the output of the signal enhancement algorithm may be used in downstream procedures, for example, to construct ab initio models. Thus, producing poor output may bias the entire computational pipeline with unpredictable consequences^(^^23^ ^)^.

### 2.4. Sorting images within classes

After removing classes of low quality, we wish to improve each of the remaining classes by removing inconsistent images. We follow the same strategy as before, and look for, within each class, a subset of images that are consistent with each other, namely, that form an approximately rank-two synchronization matrix (2.8).

Let 
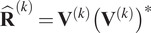
 be the best rank-two approximation of 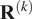 , where the columns of 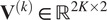 are the eigenvectors of 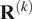 associated with the two leading eigenvalues. Let 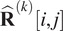 and 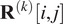 be the 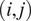 th entries of 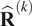 and 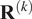 , respectively. To determine whether the 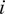 th class member is consistent with its other class members, we compute the average distance between 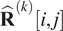 and 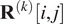 for all 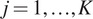 . Namely, the grade of the 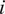 th member of the 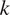 -th class is defined by
(2.10)

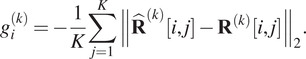

We found that producing classes with 300 images, and removing 150 images with the lowest score 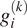 (per class) yields good results.

### 2.5. Expectation–maximization

After pruning out low-quality classes, and inconsistent images within each class, we are ready for the last stage of our algorithm: aligning the images within each class and averaging them to produce a high SNR output image. To this end, we apply the EM algorithm that aims to maximize the likelihood function of the observed images. The EM algorithm is applied to the raw images corresponding to each class separately (before down-sampling, phase-flipping, and so forth.)

The EM algorithm assumes that all observed images are rotated, translated, and noisy versions of a single image; this image is denoted by 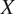 and corresponds to the high SNR image we wish to estimate. The generative model of the images within a specific class is given by
(2.11)

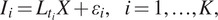

where 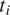 encodes the unknown rotation and translation of the 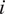 th image, 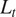 is a linear rotation and translation operator (may also include the CTF), and 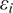 is an i.i.d. Gaussian noise with variance 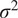 . Our goal is to maximize the marginalized log-likelihood, which is equal, up to a constant, to
(2.12)

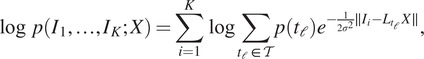

where 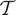 denotes the set of possible rotations and translations. While optimizing (2.12) is a challenging non-convex problem, EM has been proven to be an effective technique for optimizing (2.12) for cryo-EM images^(^^6^ ^,^^24^ ^)^. In particular, we used the implementation of RELION^(^^15^ ^)^. We note that this implementation does not correct for reflections, and thus we correct for reflections before running the EM algorithm. In particular, we construct a symmetric matrix whose 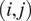 entry is −1 if the 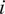 -th and 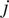 -th images are likely to be reflected, and 1 if not. This is a by-product of previous steps, described in Section 2.3, so no additional computations are required. Since this matrix is ideally a rank-one matrix (if all pair-wise estimations are consistent), we extract its leading eigenvector and round its entries into 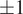 . This algorithm is known as the spectral algorithm for group synchronization^(^^22^ ^,^^25^ ^)^.

As mentioned in the introduction, a popular solution to the 2D classification task (which shares similarities with the signal enhancement problem) is to run EM on the observed images, before clustering the images. In this case, the generative model reads
(2.13)

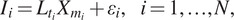

where 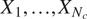 are the class averages to be estimated. While the implementation of the EM algorithm for (2.13) follows the same lines as the EM algorithm we use, it tends to output only a few informative classes because of the “rich get richer” phenomenon^(^^16^ ^)^. We evade this pitfall by first finding the nearest neighbors of the chosen images, and running EM on each class separately to optimize (2.12). In addition, since we run multiple independent instances of the EM algorithm, they can be run in parallel, resulting in a significant acceleration.

We mention that the EM algorithm can be replaced by alternative computational strategies such as stochastic gradient descent or rotationally and translationally aligning the images, and then averaging them. The latter strategy, used by^(^^13^ ^)^, is much faster than EM, and thus will significantly accelerate the algorithm, at the cost of lower image quality.

## 3. Experimental Results

In the following experiments, we produced 3000 classes and kept the best 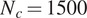 classes according to the method explained in Section 2.3. Each class consists of 300 images, from which only the best 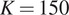 images were used to estimate the class average, as explained in Section 2.4. We used the EM implementation of RELION^(^^15^ ^)^ with seven iterations. Based on the class averages, we reconstructed ab initio models using the common-lines method implemented in the ASPIRE package^(^^26^ ^)^. All data sets were processed using an Intel(R) Xeon(R) Gold 6252 CPU @ 2.10 GHz containing 24 cores, and a GeForce RTX 2080 Ti GPU. The run times of all stages in the process are provided in Table 1. The resolution was computed based on the Fourier Shell Correlation (FSC) criterion with cutoff of 0.5, where the reference volume was downloaded from EMDB^(^^27^ ^)^.

**Table 1.** Runtime.

EMPIAR entry	# images	Dimensions [pixels]	Preprocessing [s]	sPCA [s]	Nearest neighbors search [s]	EM [s]	Total [s]
10028	105,247	360×360	1,032	1,036	131	2,299	4,508
10073	138,840	380×380	1,735	1,397	155	2,735	6,037
10081	55,870	256×256	1,405	698	170	1,149	3,427
10061	41,123	768×768	2,405	540	184	5,261	8,413

Abbreviation: EM, expectation–maximization; sPCA, steerable principal component analysis.

### 3.1. EMPIAR 10028

We begin with a data set of the Plasmodium falciparum 80S ribosome bound to the anti-protozoan drug emetine, available as the entry 10028 in EMPIAR^(^^28^ ^)^ (the corresponding entry in EMDB is EMD-2660)^(^^29^ ^)^. This data set contains 105,247 images of size 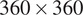 pixels.

**Figure 1.**
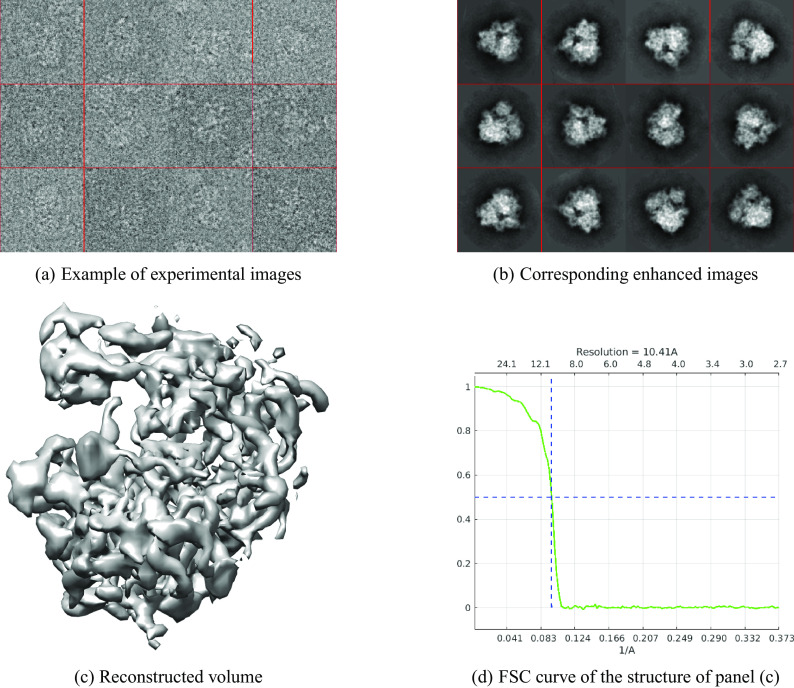
EMPIAR 10028. The resolution of the reconstructed structure is 10.41 Å.

Figure 1 shows examples of raw data images, the corresponding class averages, and a 3D structure reconstructed using the class averages; the resolution of the reconstructed structure is 10.41 Å. The nearest neighbor’s stage took 2.5 min, and the overall process took around 75 min.

### 3.2. EMPIAR 10073

**Figure 2.**
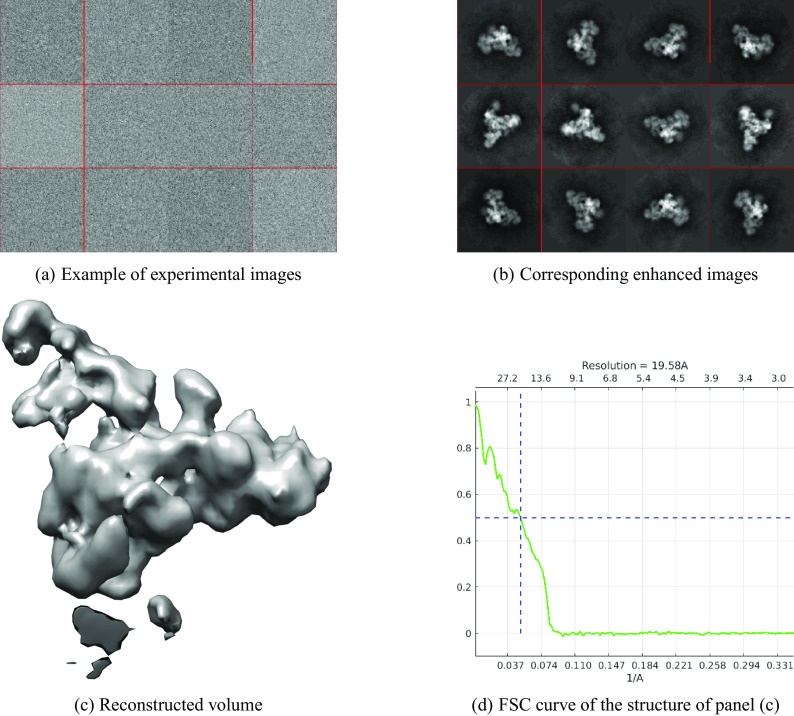
EMPIAR 10073. The resolution of the reconstructed structure is 19.58 Å.

This data set of the yeast spliceosomal U4/U6.U5 tri-snRNP is available as the entry 10073 of EMPIAR (the corresponding entry in EMDB is EMD-8012)^(^^30^ ^)^. This data set contains 138840 images of size 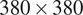 pixels. The nearest neighbors search took 2.5 min and producing 1500 class averages took roughly 100 min. The results are presented in Figure 2. The resolution of the reconstructed structure is 19.58 Å.

### 3.3. EMPIAR 10081

**Figure 3.**
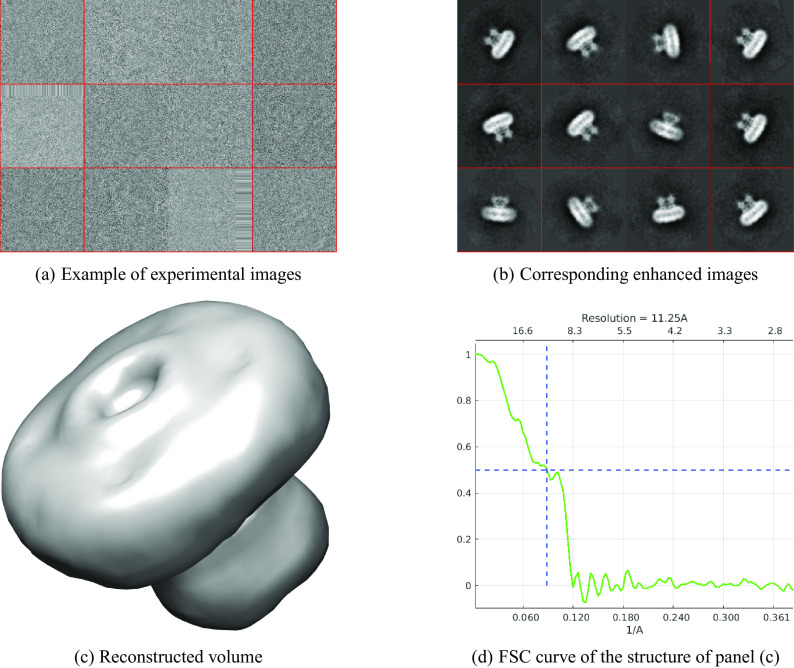
EMPIAR 10081. The resolution of the reconstructed structure is 11.25 Å.

This data set of the human HCN1 hyperpolarization-activated cyclic nucleotide-gated ion channel is available as the entry 10081 of EMPIAR (the corresponding entry in EMDB is EMD-8511)^(^^31^ ^)^. This data set contains 55870 images of size 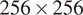 pixels. The nearest neighbors search took less than 3 min and producing 1500 high-quality images took roughly 1 hr. Figure 3 shows the results. The resolution of the reconstructed structure is 11.25 Å.

### 3.4. EMPIAR 10061

**Figure 4.**
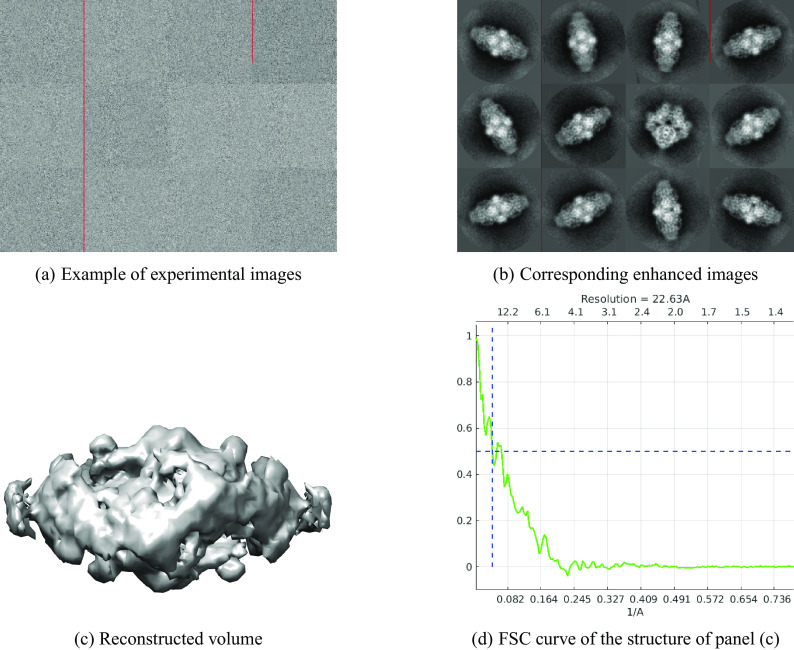
EMPIAR 10061. The resolution of the reconstructed structure is 22.63 Å.

This data set of the beta-galactosidase in complex with a cell-permeant inhibitor is available as the entry 10061 of EMPIAR (the corresponding entry in EMDB is EMD-2984)^(^^32^ ^)^. This data set contains 41123 images of size 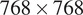 pixels. The nearest neighbors search took less than 3 min and producing 1500 class averages took roughly 2.5 hr. Figure 4 shows results, where the 3D structure was reconstructed from the enhanced images using the spectral algorithm implemented in ASPIRE^(^^33^ ^)^. Although the class averages look of good quality, the resolution of the reconstructed structure is only 22.63 Å.

## 4. Discussion

In this paper, we have presented a new algorithm to enhance the quality of cryo-EM images, which can be used for various tasks in the computational pipeline of cryo-EM. The algorithm is based on^(^^13^ ^)^, but extends it in several ways, which are crucial to improve its performance and to design built-in quality measures. The algorithm is computationally efficient and can be executed on large experimental data sets.

For larger data sets, the brute-force nearest neighbors search we use can be replaced by efficient randomized algorithms^(^^34^ ^)^, resulting in a better asymptotic computational complexity. However, for contemporary data sets, the running times of both approaches are comparable. Our classification is approximately invariant under in-plane rotations and reflections; see also^(^^35^ ^)^ for a related approach. While it can be extended to translation invariance by explicitly considering different translations, it will significantly increase the running time. A possible alternative approach would be to employ polynomials that are approximately invariant under the group of in-plane rotations and translations (namely, the group of rigid motions SE^(^^21^ ^)^.

To improve the quality of the nearest neighbors search, the classes are refined by analyzing the spectra of synchronization matrices. This provides a validation measure that can be computed directly from the data, which is crucial to mitigate the risk of model bias in downstream tasks, such as ab initio modeling. We have demonstrated that the enhanced images are of high quality so that they can be used to construct ab initio models. To cover all viewing angles, we randomly sample the data set. This is clearly not optimal, and we intend to study different deterministic strategies to sample the data. A successful sampling strategy may make the class sorting stage of Section 2.3 unnecessary.

## Data Availability

Source code and user manual are available at https://github.com/TamirBendory/CryoEMSignalEnhancement.

## Footnotes

1 We mention that there are many alternative algorithms for constructing ab initio models, which do not require enhanced images, such as stochastic gradient descent^9^ and the method of moments^10^.
